# Production of biodiesel from coastal macroalgae (*Chara vulgaris*) and optimization of process parameters using Box-Behnken design

**DOI:** 10.1186/s40064-015-1518-1

**Published:** 2015-11-24

**Authors:** Shaila Siddiqua, Abdullah Al Mamun, Sheikh Md. Enayetul Babar

**Affiliations:** Biotechnology and Genetic Engineering Discipline, Khulna University, Khulna, Bangladesh

**Keywords:** Macroalgae, *Chara vulgaris*, Algae biodiesel, Transesterification, Oil extraction, Calorific value, Yield

## Abstract

Renewable biodiesels are needed as an alternative to petroleum-derived transport fuels, which contribute to global warming and are of limited availability. Algae biomass, are a potential source of renewable energy, and they can be converted into energy such as biofuels. This study introduces an integrated method for the production of biodiesel from *Chara vulgaris* algae collected from the coastal region of Bangladesh. The Box–Behnken design based on response surface methods (RSM) used as the statistical tool to optimize three variables for predicting the best performing conditions (calorific value and yield) of algae biodiesel. The three parameters for production condition were chloroform (X_1_), sodium chloride concentration (X_2_) and temperature (X_3_). Optimal conditions were estimated by the aid of statistical regression analysis and surface plot chart. The optimal condition of biodiesel production parameter for 12 g of dry algae biomass was observed to be 198 ml chloroform with 0.75 % sodium chloride at 65 °C temperature, where the calorific value of biodiesel is 9255.106 kcal/kg and yield 3.6 ml.

## Background

One of the major priorities of every nation of the world is to provide energy security as energy is must for every sector from transportation to communication, to security and health delivery systems. Natural gas, coal and oil are the primary source of energy production. Consumption of liquid fuels around the world increases consequently from eighty-seven MMbbl/d in year 2010 to one hundred and nineteen MMbbl/d in year 2040 (US Energy Information Administration 2010). Stressing on the reduction of the energy consumption over the following decades, however, the decrease in demand of liquid fuels seems quite unrealistic.

The idea of alternative fuels generated hundred years ago, when Rudolf Diesel use peanut oil as fuel on diesel engine in 1900 world’s fair, Paris (Knothe 2001). This idea falls backwards with the increasing use of liquid fuel and natural gas, but come into focus at the end of twentieth century as the depletion of those sources in near future emerged alarmingly. In first generation biofuels, food crops are fermented to produce bioethanol, or oils collected from those crops are converted into biodiesel. Wheat or sugar crops are generally used fermentation process, whereas for biodiesel various oil seeds proved as effectual crop. Though, first generation biofuels promises to solve the fuel depletion crisis, it is associated with a number of problems. A debate arises whether it diminishes the carbon dioxide and greenhouse gas emission, because several biofuels releases more carbon to the atmosphere in comparison to their feedstock’s confine for growth. On the other hand it creates a serious controversial issue ‘fuel versus food’, since usage of food crops for fuel production shifted the supply of crops from food market to fuel market. Hence, biofuels are accused for the increasing of food prices (Naik et al. 2009).

To overcome the precincts of first generation biofuels scientists use diverse sources such as organic waste, specific biomass crops, woods, animal waste and food crop waste rather than food crops. These types of biofuels are termed as second generation biofuels and it is less costly than fossil fuels (Ralph et al. 2009). Though the second generation biofuels increase the ‘net energy gains’, but it still has some problems such as carbon dioxide and greenhouse gas emission which need to solve.

The Third Generation of biofuels is produced focusing all the problems associated with first and second generation biofuels. Rather than using food crops or crop debris it implies algae as a novel source of energy production. First of all it mostly overcomes the issue of carbon dioxide and greenhouse gas emission, as algae uptake them during the growth stage balanced these emissions. Moreover, low cultivation cost and high energy production per acre makes it more enviable source of energy. Land and water which is unsuitable for food crop production can be used to cultivate algae to diminish the use of pure water sources. From diesel to jet fuel, a broad spectrum of fuel can be manufactured from algae (Yonghua and Gilles 2013).

Several researches have been conducted worldwide on algae biodiesel. Biodiesel from microalgae (Chisti 2007); Algae-based biofuels: applications and co-products (Iersel and Flammini 2010); Opportunities and challenges in algae biofuels production (Benemann 2008); A realistic technology and engineering assessment of algae biofuel production (Lundquist et al. 2010); Combustion analysis of algal oil methyl ester in a direct injection compression ignition engine (Hariram and Kumar 2013). Macroalgae use sunlight energy since they are photosynthetic organism, consume more carbon dioxide than the conventional food crops and produce huge amount of biomass per area. Macroalgae could be an immense source of biofuels production as it grows in large amount in fresh water, slightly brackish water to the marine water around the world.

*Chara vulgaris* is a macroalgae grown in large quantities in the brackish water of coastal region of Bangladesh. This research work is focused on the oil extraction and biodiesel production from *Chara vulgaris*. Chemical process was applied for oil extraction and transesterification process. Chloroform, methanol, sodium chloride and temperature are significant factors for chemical method of algae biodiesel production. For optimization of chemical method of algae biodiesel production Box–Behnken design was used. The Box–Behnken design (Box and Behnken 1960), which is the response surface methods (RSM), is a very useful statistical tool to optimize multiple variables for predicting the best performing conditions by using a minimum number of experiments (Babar et al. 2007).

## Methods

### Materials

*Chara vulgaris* was collected from southern part of Khulna, Bangladesh, chosen on the basis of higher accessibility. It is found throughout the year but higher amount found from May to September. Total lipid content of *Chara vulgaris* is 73.2 mg/g of dry weight (Dembitsky and Rezanka 1993). Total lipid extraction from algae performed by chemical extraction methods, relying on the chloroform–methanol solvent system, based on the Bligh and Dyer method (Bligh and Dyer 1959). Sodium chloride solution was added to facilitate oil extraction from algae (Axelsson and Gentili 2014). A slight modification of this process was done to facilitate the lipid extraction from *Chara vulgaris*. Lipid found from this step was then used for the transesterification reaction to produce biodiesel. Chloroform (Loba Chemie, Mumbai, India), Sodium Chloride (Merck, Mumbai, India), Methanol (Merck KGaA, Darmstadt, Germany) and Sodium hydroxide (Merck, Mumbai, India) used for the oil extraction and transesterification process.

### Oil extraction

Debris was separated from *Chara vulgaris* followed by washing with running water and distilled water. Paste of *Chara vulgaris* was prepared by using mortar and pestle and then dried in the incubator at 80 °C for 30 min. 12 g of dry algae biomass was mixed with chloroform and methanol, shake vigorously for 10 min. Then NaCl solution added in the mixture and shake for another 10 min. Resulted mixture was filtered to remove the algal biomass. The clear solution kept for a while to form two distinct phases. The upper phase which contains water and methanol are separated from the lower chloroform-oil phase and discarded. The lower chloroform-oil phase was evaporated to remove excess chloroform and the remaining solution was used for the further treatment.

### Biodiesel production

Alkaline catalyst sodium hydroxide, at an amount of 0.8 % of algal oil was dissolved in the methanol at an amount of 25 % of algal oil by hand shaking and whirling. Resulting sodium methoxide was then added to the preheated (at 50 °C) algal oil and air tight the reaction system to avoid the loss of methanol. Temperature was kept constant at 55–65 °C and heated for 3 h to complete the reaction. Once the reaction was completed, two major products exist-biodiesel and glycerin. Biodiesel was separated from glycerin by gravity settling as the glycerin was much denser than biodiesel, it settled down at the bottom.

Separated biodiesel contained some soap and methanol. The methanol was removed by vaporization. After the methanol had been removed, the biodiesel was washed with distilled water by liquid–liquid extraction process to remove the soap, and catalyst. The washing procedure was repeated for 3–4 times until the soap totally removed. Remaining water present in the biodiesel was removed by heating it at 100 °C for 10 min. Finally usable form of biodiesel was found (Mamun et al. 2013).

### Properties determination of biodiesel

The calorific value of algae biodiesel was determined by ‘oxygen bomb calorimeter’. Kinematic viscosity and flash point were determined by ‘SAYBOLT/REDWOOD viscometer bath’ and ‘flashpoint tester, type-00-ESR’ respectively. Density was determined by ‘mass/volume’ equation.

### Experimental design

For the optimization of process parameters of algae biodiesel production a very popular statistical tool was applied. Box-Behnken design is an independent quadratic design and requires three levels of each factor (Bligh and Dyer 1959). For three factors, it offers some advantage by entailing a smaller number of runs for three factors. The important factors for the production were chloroform, NaCl and temperature and the three levels are +1, 0 and −1 correspondingly. Hence, these factors were considered as the independent variables and their effects on calorific value and yield of algae biodiesel were studied using Box-Behnken design of Response surface methodology (RSM).

The range and levels of experimental variables investigated in this study were presented in Table 1. The central values (zero level) chosen for experimental design were: chloroform-X_1_ (158.4 ml i.e. 13.2 ml chloroform/gm of wet algae biomass), NaCl-X_2_ (0.75 %) and temperature-X_3_ (60 °C). Chloroform level was calculated at the zero level by maintaining the 2:1 ratio of chloroform: methanol.

**Table 1:** Coded levels of independent variables in the experimental design

Variables*	Coded levels		
	+1	0	−1
Chloroform (ml)	118.8	158.4	198
NaCl (%)	0.70	0.75	0.80
Temperature (°C)	55	60	65

*Where, each of chloroform and NaCl concentration is given for 12 gm of dry algae biomass

The second order polynomial equation was used to produce predicted value to facilitate the production of surface plot. The second order polynomial equation uses regression coefficient for the analysis. The second order polynomial equation for three responses:

1 $$Y = A_{0} + A_{1} X_{1} + A_{2} X_{2} + A_{3} X_{3} + A_{4} X_{1} X_{2} + A_{5} X_{1} X_{3} + A_{6} X_{2} X_{3} + A_{7} X_{1}^{2} + A_{8} X_{2}^{2} + A_{9} X_{3}^{2}$$

where Y is the response; X_1_ chloroform, X_2_ NaCl concentration, X_3_ temperature; A_0_ the regression coefficient, A_1_– A_3_ are the linear coefficients, A_4_–A_6_ the cross product coefficients, and A_7_–A_9_ are the quadratic coefficients. The regression analysis, statistical significance and ANOVA were carried out using Microsoft Office Excel. Surface plots and contour plot were developed using the same software along with Sigma Plot software.

## Result and discussion

### Production process optimization

Lipid from *Chara vulgaris* was extracted by using chloroform, methanol and sodium chloride solvent. Sodium chloride acts as an inhibitory agent between the binding of acidic lipids and denatured lipids during lipid extraction from algae. Crude lipid extract was then transesterified for 3 h by methanol and sodium hydroxide at 55–65 °C temperature.

Important physical properties of algae biodiesel produced from *Chara vulgaris* were determined and it was found that values were resided within the ASTM range of the physical properties of biodiesel presented in Table 2. The value of viscosity of algae biodiesel was 5.003 centistokes that reside within the American Society for Testing and Materials (ASTM) standard of biodiesel. Higher flash point and calorific value indicate the high quality biodiesel; flash point and calorific value were found 133 °C and 9255.106 kcal/kg correspondingly exist within the (ASTM) standard. Density value was also within the (ASTM) standard of biodiesel.

**Table 2:** Physical properties of algae biodiesel produced from *Chara vulgaris*

Properties	Algae biodiesel	Biodiesel (ASTM)
Viscosity	5.003 cst	3.7–5.8 cst
Flashpoint	133 °C	>130 °C
Calorific value	9255.106 kcal/kg	8850–10,000 kcal/kg
Density	0.871 g/ml	0.87 to 0.89 g/ml

The Box-Behnken design for three independent variables, i.e. chloroform, NaCl and temperature and the experimental values of calorific value and yield found by using the corresponding design are shown Table 3. These values were used in the regression analysis where confidence level was kept at 95 % to get the coefficients for each response.

**Table 3:** The Box-Behnken design matrixes employed for *Chara vulgaris* biodiesel

Run no.	Chloroform (X_1_) (ml)	NaCl (X_2_) (%)	Temperature (X_3_) (°C)	Lipid extract before transesterification (ml)	Calorific value (c) (kcal/kg)	Yield (y) (ml)
1	118.8	0.70	60	14	9167.173	3.0
2	198	0.70	60	18	9153.926	3.4
3	118.8	0.80	60	13	9108.31	2.9
4	198	0.80	60	19	9219.738	3.4
5	118.8	0.75	55	15	9188.192	3.1
6	198	0.75	55	20	9242.961	3.6
7	118.8	0.75	65	14	9132.539	3.0
8	198	0.75	65	19	9255.106	3.5
9	158.4	0.70	55	17	9158.281	3.2
10	158.4	0.80	55	16	9151.03	3.1
11	158.4	0.70	65	17	9143.935	3.2
12	158.4	0.80	65	17	9174.205	3.2
13	158.4	0.75	60	18	9169.471	3.3
14	158.4	0.75	60	18	9169.471	3.3
15	158.4	0.75	60	18	9169.471	3.3

Where, each of chloroform and NaCl concentration is given for 12 g of dry algae biomass

The *p*-values shown in the Table 4 are indicator of the interaction strength between each independent variable and a *p* value less than 0.05 indicate that the factor interacted significantly with the response. It is observed that all the *p* values are smaller than 0.05, indicates that all linear factor, cross product factor and quadratic factor significantly related to calorific value except one cross product factor and one quadratic factor like temperature. It is seen that chloroform-NaCl cross product are significantly related to the response (c). Though yield does not show significant relation with chloroform and temperature, but significantly related to the linear factor and quadratic factor like NaCl. R^2^ value is found by the ANOVA analysis for calorific value and yield are 0.947995 and 0.976573 respectively.

**Table 4:** Regression coefficient and corresponding probability values (*p*-values) for specific responses (calorific value and yield)

Parameter (coefficient)	Calorific value (c)		Yield value (y)	
	Coefficient	*P*-value	Coefficient	*P*-value
Constant (A_0_)	9680.969	0.01485	−19.25	0.078605
X_1_(A_1_)	−20.1792	0.004058	−0.00347	0.803372
X_2_(A_2_)	11836.46	0.069057	66.5	0.010884
X_3_(A_3_)	−114.098	0.051354	−0.0775	0.620817
X_1_ X_2_(A_4_)	15.74179	0.009388	0.012626	0.363217
X_1_ X_3_(A_5_)	0.085604	0.076392	−2.6E−18	1
X_2_ X_3_(A_6_)	37.521	0.272441	0.1	0.363217
X_1_ X_1_(A_7_)	0.012962	0.050241	1.12E−19	1
X_2_ X_2_(A_8_)	−11004.2	0.017777	−50	0.004867
X_3_ X_3_(A_9_)	0.59609	0.118613	2.25E−17	1

From regression analysis all nine coefficients are used in making the response equation, though not all the factors are significant (p < 0.05). The second-order polynomial equations for calorific value and yield are given below respectively:

2 $$Y(c) = 9680.969 - 20.1792X_{1} + 11836.46X_{2} - 114.098X_{3} + 15.74179X_{1} X_{2} + 0.085604X_{1} X_{3} + 37.521X_{2} X_{3} + 0.012962X_{1}^{2} - 11004.2X_{2}^{2} + 0.59609X_{3}^{2}$$

3 $$Y\left( y \right) = - 19.25 - 0.00347X_{1} + 66.5X_{2} - 0.0775X_{3} + 0.012626X_{1} X_{2} - \left( {2.6E - 18} \right)X_{1} X_{3} + 0.1X_{2} X_{3} + \left( {1.12E - 19} \right)X_{1}^{2} - 50X_{2}^{2} + (2.25E - 17)X_{3}^{2}$$

where Y(c) is calorific value and Y(y) yield value, X_1_, X_2_ and X_3_ are coded values for chloroform, NaCl and temperature correspondingly.

### Response surface analysis

**Fig. 1:**
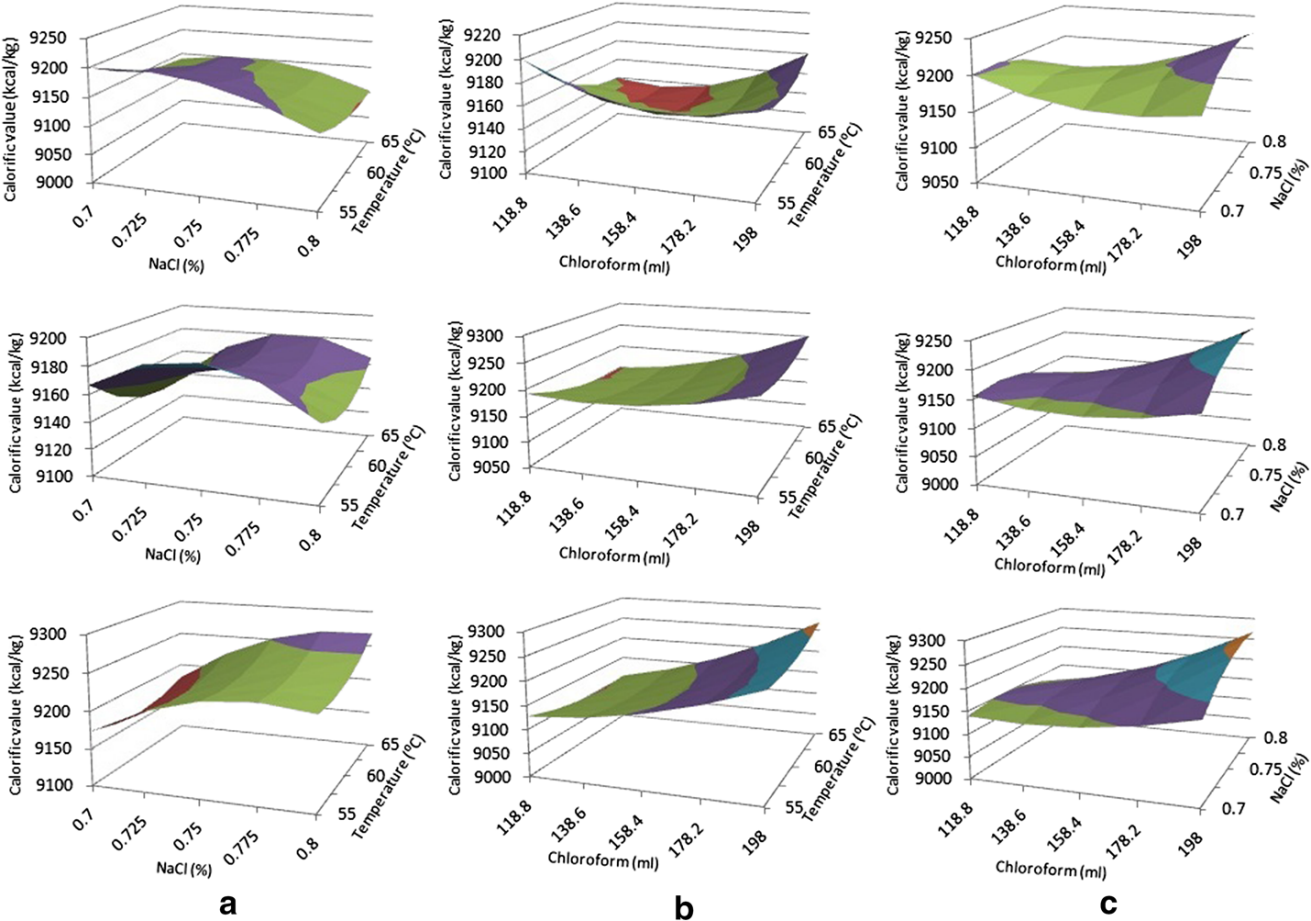
Response surface plot for all design condition. **a** Effects of temperature and NaCl on calorific value at constant chloroform (ml), **b** Effects of temperature and chloroform on calorific value at constant sodium chloride concentrations, and **c** Effects of chloroform and NaCl concentrations on calorific value at constant temperature (°C)

**Fig. 2:**
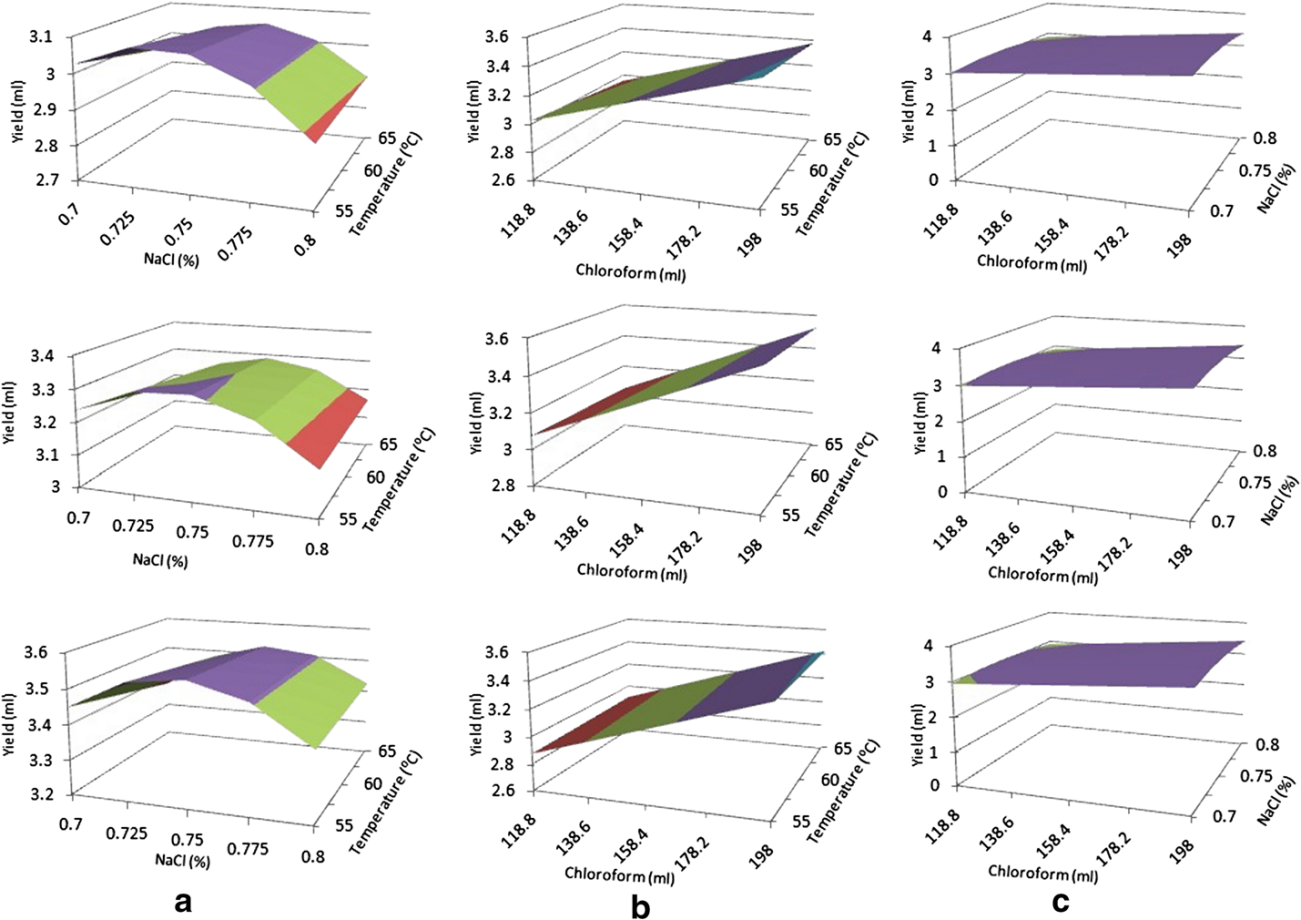
Response surface plot for all design condition. **a** Effects of temperature and NaCl on yield at constant chloroform (ml), **b** Effects of temperature and chloroform on yield at constant sodium chloride concentrations, and **c** Effects of chloroform and NaCl concentrations on yield at constant temperature (°C)

The effect of three variables on the response is seen by holding one factor constant while the other two factors are varied. The values of calorific value and yield are predicted by using Eqs. (2) and (3) and all the 3-D response surface plots are shown in Figs. 1 and 2. Due to three coded levels and the three coded variables total nine combinations are possible for each response. The plots are created with the aim to observe optimum condition from predicted values.

When chloroform is kept constant at 198 ml the surface plot for calorific value showed a higher point at the corner at 0.75 % NaCl (Fig. 1a). This state produced a good calorific value, both in experiment and prediction. As high calorific value indicates better quality biodiesel, the plot is observed on this basis. However, the calorific value is found much lower for the low level of chloroform. Figure 1b can be helpful in understanding the patterns of NaCl concentration. It can be seen that, high calorific value observed at the middle of NaCl concentration with increase Chloroform concentration and temperature. From this curve, it can be observed that the pattern of responses varies with respect to chloroform and temperature. When the responses are varied with three factors the relationship became more complex, that is why low value was observed at 0.70 %, high value at 0.75 %, and low value again at 0.80 % NaCl concentration (Fig. 1a, b). From this, it can be concluded that at 0.75 % NaCl concentration calorific value is optimum with varying chloroform concentration and temperature. As shown in the Fig. 1c similar patterns are found for varying temperature factor. All the patterns show low response at low temperature and increases with temperature. Similar results can be seen from Table 3, which contains experimental values. It also found that when temperature is stay constant at 65 °C (Fig. 1), maximum calorific value 9255.106 kcal/kg is observed at 198 ml chloroform and 0.75 % NaCl. This condition is considered as optimum and thus used for further study.

Similar 3-D surface plots are created to see the effect of the three variables on the yield response. From Fig. 2a, b it can be perceived that yield increases with increasing chloroform quantity. High response is observed at 198 ml chloroform concentration. Whereas high yield found at the middle point of the NaCl concentration at 0.75 %, low response found in both low level and high level of NaCl concentration correspondingly at 0.70 and 0.80 %. But the effects of different temperature on the response are varied very slightly. From this it can be calculated that NaCl concentration has much more effect on the response rather than chloroform and temperature. Higher response found at 0.75 % NaCl concentration, 198 ml chloroform and at 65 °C temperature, which is similar found in experimental value. Thus, this is the optimum condition for yield of algae biodiesel.

### Contour plot analysis

**Fig. 3:**
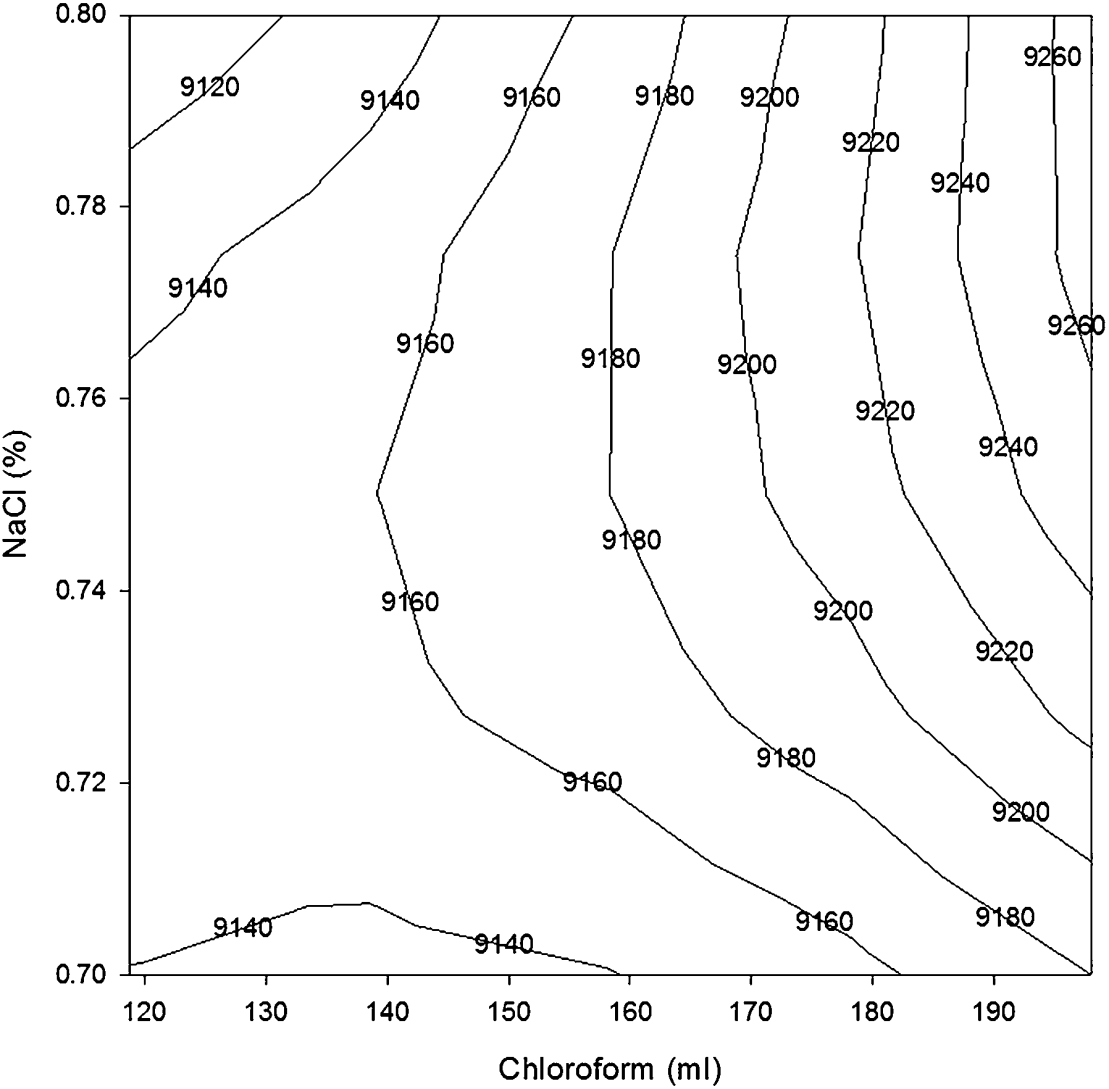
Contour plot showing calorific value at various NaCl concentrations and chloroform at constant 65 °C temperature

**Fig. 4:**
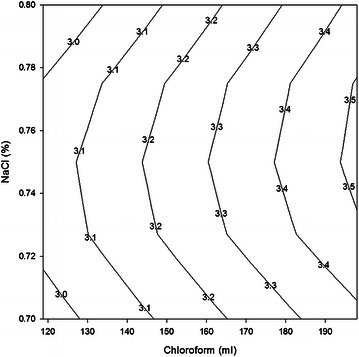
Contour plot showing yield at various NaCl concentrations and chloroform at constant 65 °C temperature

The contour plot is produced by plotting NaCl concentration (Y axis) against chloroform (X axis) for a series of predicted value at a constant temperature of 65 °C. Figures 3, 4 stands for the contour curve for calorific value and yield correspondingly where different values expressed a diverse zone.

Experimentally, only one combination produced maximum calorific value (Table 3). From the curve, it is found that several combinations of NaCl concentrations and chloroform are likely to produce the same line. It can be observed that an extremely smaller portion on the curve in right corner consists of high calorific value (9260 kcal/kg) zones and smaller lower calorific value (9120 kcal/kg) zone at the left corner. It is found that a higher calorific value zone could be obtained using a NaCl concentrations range of 0.76–0.80 % and chloroform range of 196–198 ml, while a lower calorific value zone resulted from a NaCl concentrations range of 0.78–0.80 % and chloroform range of 120–130 ml. This curve signifies that high calorific value is more responsive to NaCl concentrations than to chloroform when temperature is kept constant.

Figure 4 demonstrates that a very smaller portion on the curve in right edge consists of high yield (3.5 ml) zones and smaller lower yield (3.0 ml) zone at the lower left corner. It is found that a higher calorific value zone could be obtained using a NaCl concentrations range of 0.73–0.77 % and chloroform range of 196–198 ml. While a lower calorific value zone resulted from a NaCl concentrations range of 0.70–0.71 % and chloroform range of 120–128 ml. This curve point out that high calorific value is more responsive to NaCl concentrations than to chloroform when temperature is kept constant.

The process is validated by random selection of few values from the design combination. Experimental and predicted values of calorific value and yield are shown in the Table 5 reveal that they are quite close. This research validated that chloroform; NaCl concentration and temperature are critical factor for algal biodiesel production.

**Table 5:** Experimental and predicted calorific values for method validation experiment

Chloroform (ml)	NaCl (%)	Temperature (°C)	Calorific value (kcal/kg)		Yield (ml)	
			Experimental	Predicted	Experimental	Predicted
118.8	0.7	55	9116.294	9147.441	3.1	3.0649
118.8	0.8	65	9123.083	9114.326	3	2.9529
158.4	0.75	65	9157.839	9176.299	3.2	3.2610
198	0.8	65	9204.372	9222.489	3.4	3.4201

## Conclusion

Biodiesel was produced from macroalgae *Chara vulgaris* and the three production parameters were optimized using Box–Behnken design to predict the best performing conditions for calorific value and yield of algae biodiesel. The three parameters for production condition were chloroform, sodium chloride concentration and temperature. Optimal conditions were estimated by the aid of statistical regression analysis and surface plot chart. The Optimal condition of biodiesel production parameter was recognized to be 198 ml Chloroform with 0.75 % sodium chloride at 65 °C temperature where the calorific value of biodiesel was 9255.106 kcal/kg and yield 3.6 ml. This study signifies that the biodiesel produce from *Chara vulgaris* could be used as a possible alternative fuel and further study will make it suitable for large scale production.
